# Book Reviews

**Published:** 1895-05

**Authors:** 


### Book Reviews.

A SYSTEM OF LEGAL MEDICINE. By ALLAN MCLANE HAMILTON, M.D., Consulting Physician to the Insane Asylums of New York City, etc., etc., and Lawrence Godkin, Esq., of the New York Bar. With twenty-seven collaborators. Illustrated. Volume II. Large 8vo, pp. 738. Price per volume, cloth, $5.50 ; full sheep, $6.50. New York: E. B. Treat, 5 Cooper Union. 1894.

The second volume of this important work introduces itself with a chapter relating to the duties and responsibilities of medical experts, written by William B. Hornblower. This is a subject of much interest, is ably handled by the distinguished author and could have been considerably extended with much propriety.

The next chapter, a lengthy one, but divided into parts and subsection, is written by the medical editor-in-chief and is devoted to the consideration of insanity in its medico-legal bearings. Dr. Hamilton deals with his subject exhaustively as well as ably. This might be expected from the distinguished author's well-known skill and training as an alienist. This is a broad subject with many collateral relations, some of which are exceedingly intricate and difficult to differentiate as well as to understand. This section is supported by a long bibliographical list and index. Next comes a brief chapter by Judge Calvin E. Pratt on the mental responsibility of the insane in civil cases. This short article is full of interesting statements and references.

Insanity and crime form the subject of the next chapter, written by Dr. B. Sachs. Here is found a field for extended observation and comment that the author has occupied to good advantage. Dr. Sachs comments on the case of Guiteau in a manner which, to us, seems supremely silly. To declare at this late date that Guiteau was a pronounced lunatic, yet was hanged to satisfy the public conscience, and this in spite of the fact that he had a careful and over-patient trial, prolonged beyond reason, and attended by the ablest alienists in the land, is, to our mind, an unnecessary display of sympathy with a bad cause.

The next chapter deals with the relations of mental defect and disease to criminal responsibility and is written by Louis E. Binsse. This section contains much that is instructive and gives the law in the several states bearing on the questions that it discusses.

Aphasia and other affections of speech is made the subject of another interesting chapter by Charles K. Mills. The medico-legal aspects of aphasia furnish a curious and somewhat complicated entanglement of law and medicine, but it is ably handled by the distinguished author. One of the most important subjects dealt with in the volume is the traumatic neuroses by Charles L. Dana. This is a description of the chronic nervous disorders that follow shock and injury. It possesses special interest to railway surgeons and ought to be studied carefully by every physician who assumes charge of an injured patient, where there is likely to arise a claim for damages. Eight and a half pages of bibliography follow this section, which indicates its importance in the literature of medicine.

The next chapter considers the effects of electric currents of high power upon the human body and is written by Allan McLane Hamilton and George DeForest; then follows a short chapter on accident cases, by Lawrence Godkin; next, one on mental distress as an element of damage in cases to recover for personal injuries; then one on feigned diseases of the mind and nervous system, by Philip Coombs Knapp. This latter chapter is one of great importance to medical experts who appear in court in relation to alleged diseases and injuries resultant from accidents, where claim is made upon railway companies, municipal corporations and life or accident insurance companies. Malingering is often difficult of detection. It requires all the tact and acumen that skill and experience can furnish and then frequently escapes undetected.

An important section follows, devoted to birth, sex, pregnancy and delivery, which are ably discussed in all their medico-legal bearings, by Andrew F. Currier. In this section are developed some of the most important duties of the medical witness, their importance being in direct ratio to their delicacy. Abortion and infanticide form the subjects of the next section, which is written by Charles Jewett. Here again occur questions that often overtax the medical witness in his efforts to separate the false from the true. Genito-urinary and venereal affections in their medico-legal relations are next considered by F. R. Sturgis. This author always writes intelligently, concisely and instructively; never more so, perhaps, than in this instance. Marriage and divorce appropriately follow and are considered by Simeon E. Baldwin in a short but pithy section. Sexual crimes form the subject in the next section, which is treated of by Charles Gilbert Chaddock. The literature on this subject has greatly increased of late and the author of this chapter may be considered an expert on the subject. He writes intelligently and plainly upon a delicate subject, elevating it from disgust to science.

Surgical malpractice forms the concluding section of the book and is ably handled by George Ryerson Fowler. This distinguished surgeon and able writer has written something of interest to every physician called upon to treat surgical disease or injury.

A concise epitome of the laws relative to the insane has been made and published by H. P. Stearns and is added as an appendix to this book by his permission. An elaborate index covering both volumes, of fifty-eight pages length, closes the volume.

We have done very imperfect justice to this valuable treatise. We have not sought to analyze its work, but rather to point out its scope and indicate its importance. Medical jurisprudence has moved forward somewhat rapidly within the past few years, though it is proper to add that until lately it had not kept pace with other departments of medical science. We believe it to be a work that will contribute to further advance the science of forensic medicine.

---

Twentieth Century Practice. An International Encyclopedia of Modern Medical Science. By Leading Authors of Europe and America. Edited by Thomas L. Stedman, M. D., New York City. In twenty volumes. Volume I. Diseases of the Uropoietic System. New York: William Wood & Co. 1895.

The trend of medical literature is toward the writing and publishing of monographs. This is in obedience to a demand following a taste cultivated in the direction named, growing out of the tendency toward the practice of specialties. Even the old-fashioned work on the practice of medicine is subdivided and split up so that modern treatises on general practice do not cover the same ground or spread over the extent of territory that they did a quarter of a century ago. The teacher of medicine in the schools yields up to his colleagues in the several branches many of the subjects that he formerly felt called upon to discuss in the lecture room.

Since the appearance of Ziemssen's encyclopedic work about 1875, which is really a group of monographs on internal medicine, all works on practice have followed the trend that we have indicated above. The same publishers who brought out the English translation of Ziemssen are now accentuating the closing years of the nineteenth century by issuing a new cyclopedia of modern medicine, which it is proposed shall be an embodiment of the science of internal medicine as enriched by the most modern studies in chemistry, pathology and bacteriology. The laboratories of the greatest teachers of these sciences may well be made to yield everything that they contain of value, up to the last moment when each volume goes to press. The work will consist of twenty volumes, the first twelve being devoted to systemic affections, including diseases of the skin and nervous system; while the last eight will be devoted to a consideration on the infectious diseases.

The first volume of this remarkable work is before us, and a careful examination of its contents justifies the assertion that the publishers have not promised too much in their prospectus. We have not space at our command at present to give the book an analytical review, nor indeed, had we the space, would it be necessary to do so. A simple statement of the contents of the volume, together with the names of the authors who discourse upon the several subjects treated of, is quite sufficient to give our readers all information needful to furnish a basis for a decision in reference to its purchase.

The first article, on diseases of the kidneys, is from the pen of Dr. Francis Delafield, of New York. The classification of kidney diseases which the author makes is extremely simple, and assists the reader greatly in arriving at a clear understanding of the morbid changes which these organs undergo. The diseases of the renal pelves, the ureters and the bladder are presented in two excellent articles, by Mr. Reginald Harrison, London. These are followed by two systematic and lucid treatises on the diseases of the prostate and male urethra, by Dr. G. Frank Lydston, of Chicago. The diseases characterized by changes in the urine (hematuria, cystinuria, chyluria, pyuria, etc.,) are discussed by Mr. E. Hurry Fenwick, of London, an acknowledged authority on these affections. The albuminurias of nephritis and diabetes mellitus are not included in this article. The closing treatise of the volume is one on the diseases of the female bladder and urethra, by Dr. Howard A. Kelly, of Baltimore. In this article the author describes at length his new method of examination of the bladder and ureters in the female, which, we believe, has never before been described in any text-book or treatise.

The illustrations, of the first and last articles especially, are beautiful in design and execution.

It may seem at first somewhat odd to call a treatise, based upon the experiences of the present century, the Twentieth Century of Practice, but it is only a name after all and may serve as well as any other.

---

Obstetric Surgery. By Egbert H. Grandin, M. D., Obstetric Surgeon to the New York Maternity Hospital; Gynecologist to the French Hospital, etc.; and George W. Jarman, M. D., Obstetric Surgeon to the New York Maternity Hospital; Gynecologist to the Cancer Hospital, etc. With eighty-five illustrations in the text and fifteen full-page photographic plates. Royal 8vo, pp. 220. Extra cloth, $2.50 net. Philadelphia: The F. A. Davis Co., Publishers, 1914 and 1916 Cherry street. 1894.

The practice of obstetric surgery has only lately taken its place as a science. Ten or twelve years ago it was limited to a few mechanical operations that were very often unsuccessful because of want of attention to proper aseptic and antiseptic details. Now, thanks to the improved technique resulting from a better understanding of the principles of clean surgery, there is almost no limit to the successful use of the knife in the lying-in chamber. Every obstetrician must be prepared to do a Sänger or Porro-Cesarean section, symphysiotomy or any other emergency operation.

The necessity for this sort of instruction imposes the duty of preparing text-books devoted especially to surgical obstetrics. The volume before us is a pioneer in this kind of literature. It possesses many valuable points. The introductory chapter, devoted to asepsis and antisepsis, is very good, but might have been much extended with propriety. Obstetric dystocia and its determination is well handled in Chapter I. Especially to be commended are the illustrations giving the different varieties of deformed pelvis. All the lesser degrees of obstetric surgery, which may be called minor, such as artificial abortion, the induction of premature labor, application of the forceps and the employment of version are very well handled; but the major procedures in surgical obstetrics—namely, symphysiotomy and Cesarean section, are hardly up to the standard we might expect in such a work. The authors have hardly risen to the importance of their subjects in this department. These chapters should have been written by practical surgeons, men who have opened the abdomen hundreds of times, and especially by an author who has made hysterectomy a study and who has successfully performed Cesarean section a number of times, and who can point to a series of completed Porro operations without undue mortality. We hope to see future editions of this work improved in respect to the consideration of major surgical operations in obstetrics.

---

DIFFICULT LABOR: A guide to its management, for Students and Practitioners. By G. ERNEST HERMAN, M. B., Lond., F. R. C. P., Senior Obstetric Physician to the London Hospital; Physician to the General Lying-in Hospital; President of the Obstetrical Society of London; Examiner in Midwifery to the Royal College of Surgeons; Late Physician to the Royal Maternity Charity. Crown octavo, 442 pages, 162 illustrations. Price, muslin, $2.25. New York: William Wood & Company. 1894.

There is no more important subject connected with obstetrics than that of the management of difficult labor, nor is there one on which the student or young practitioner stands in greater need of practical instruction. No matter how thorough teachers may be, there is always need of some safe book of reference that may guide during the early years of practice. The text-books of the period are inadequate to meet the requirements of a higher and more perfected order of obstetrical education on this point. They perplex the learner by telling him too many things that he may do without informing him which is best. There is every reason why this branch of obstetrics should receive special treatment in a separate treatise by an experienced obstetrician.

The author of this work has had just such an experience, one larger than that of most persons, in the management of difficult labors, which fits him in an eminent degree to give practical instruction on the subject named. He does not render the treatise cumbersome by describing the many different things that may be done, but he tells us clearly and directly what he thinks is best to do in dealing with each complication of labor, and the reasons why he thinks so.

The book is concisely written, dogmatic in style, but not unduly so when the object of the writer is considered, and is well illustrated. It should be found within easy reach of every man who attempts the practice of obstetrics, while teachers will find it vastly useful in aiding to methodize their instruction. Colleges will, of course, adopt it as one of the text-books in the department of obstetrics.

---

The Principles of Surgery and Surgical Pathology. General Rules Governing Operations and the Application of Dressings. By Dr. Hermann Tillmanns, Professor in the University of Leipzig. Translated from the third German edition by John Rogers, M. D., New York, and Benjamin Tilton, M. D., New York. Edited by Lewis A. Stinson, M. D., Professor of Surgery in the University of the City of New York, Medical Department. With 441 illustrations. Octavo, pp. viii.—800. New York: D. Appleton & Co. 1894.

This author strikes right into the subject of his treatise in the first chapter of his book, in which he gives minute directions for the preparation of an aseptic surgical operation; and in his next chapter he considers anesthesia with much elaboration. These two chapters deserve careful study.

It was a wise combination of subjects in considering the principles of surgery and its pathology in the same treatise. It enables the surgeon to refer to both branches of the subject without loss of time and each serves to accentuate the importance of the other. Not since Billroth's classic treatise on surgical pathology, that appeared some twenty-three years ago, has there been a more satisfactory exposition of surgical pathology than here given by Tillmanns. It is brought down to the immediate present under the light afforded by the most modern researches in bacteriology. A student should be taught pathology before he is instructed in surgical diseases and injuries. These latter he will then understand with a clearness that could not be possible if the method of teaching were reversed.

The editor and the translators appreciating this fact have duly emphasized it in bringing out and making available as a text-book one of the best treatises on the principles of surgery and surgical pathology that has yet been written.

It is impossible in the space now at our disposal for us to do more than express our opinion of this excellent work and to commend it to student and practitioner as a safe and scientific guide, which we do here and now.

---

INTERNATIONAL CLINICS. A Quarterly of Clinical Lectures on Medicine, Neurology, Surgery, Genito-urinary Surgery, Gynecology, Pharyngology, Rhinology, Ophthalmology, Laryngology, Obstetrics, Otology and Dermatology. By professors and lecturers in the leading medical colleges of the United States, Great Britain and Canada. Edited by JUDSON DALAND, M. D. (Univ. of Penna.), Philadelphia, Instructor in Clinical Medicine and Lecturer on Physical Diagnosis in the University of Pennsylvania; Assistant Physician to the University Hospital; Physician to the Philadelphia Hospital and Fellow College of Physicians, Philadelphia. J. MITCHELL BRUCE, M. D., F. R. C. P., London, England, Physician to, and Lecturer on, Therapeutics at the Charing Cross Hospital. DAVID W. FINLAY, M. D., F. R. C. P., Aberdeen, Scotland, Professor of Practice of Medicine in the University of Aberdeen; Physician to, and Lecturer on, Clinical Medicine in the Aberdeen Royal Infirmary; Consulting Physician to the Royal Hospital for Diseases of the Chest, London. Volume IV. Fourth series. 1895. Royal 8vo, pp. x.—365. Philadelphia: J. B. Lippincott Co. 1895.

The only Buffalo contributor to this volume is Dr. Matthew D. Mann, who lectures on gonorrheal salpingitis.

Beside this, we would call attention to a lecture on mania, by Dr. George H. Rohé, of Baltimore, and one on forms of insanity, by D. C. B. Burr, of Flint, Mich.

A well-illustrated lecture, that possesses considerable interest, on lateral curvature of the spine, is given by Dr. Reginald H. Sayre, of New York. Sir Dyce Duckworth, of London, presents an instructive lecture on jaundice from obstruction of the common bile duct. A number of illustrated surgical lectures appear in this volume, which is uniform with its predecessors in size, type and binding, and closes the fourth series.

---

A COMPEND OF THE PRACTICE OF MEDICINE. By DAVID E. HUGHES, M. D., Chief Resident Physician Philadelphia Hospital; Physician-in-Chief, Insane Department, Philadelphia Hospital, etc., etc. Fifth Physicians' edition, thoroughly revised and enlarged. Including a very complete section on skin diseases and a new section on mental diseases. Small 8vo; pp. viii.—568. Price, $2.50. Philadelphia: P. Blakiston, Son & Co., 1012 Walnut street. 1895.

The demand for this work in the past has been sufficient to exhaust four editions. Hence it may be considered as having an established place in the literature of medicine. There is no doubt but that the present methods of teaching are such as to demand the use of compends, and this one is most excellent in its plan and consideration of the subjects that belong to the practice of medicine. It was originally designed as an aid to students, to be used, of course, in connection with larger treatises, but having outgrown its original purpose physicians may also find it a useful reference book.

In this fifth edition there has been a thorough revision, enabling the author to incorporate recent discoveries in medicine and add a section on mental diseases. The book is beautifully printed, is bound in black morocco with flexible covers and gilt edges, making it one of the handsomest medical books in appearance that we have seen.

---

Transactions of the Colorado State Medical Society. Twenty-fourth Annual Convention. Denver, June, 1894. Published for the society.

Though the Medical Society of the State of Colorado is but twenty-three years old, it publishes a volume of transactions that many an older state might envy. The material is excellent and the mechanical make-up of the volume is beyond criticism. Some of the subjects dealt with are unique and possess particular interest at this time. The new woman comes in for her share in the way of two papers that not only could she read with interest, but others who are studying into the causes and prevention of disease. Dr. Mary E. Bates, of Denver, read a paper on the new movement in dress reform and exhibited a number of women and children attired in this new costume.

Bicycle exercise for women was the title of a paper presented by Dr. Laura L. Liebhardt, of Denver. The value of the two papers could have been much enhanced by well-drawn illustrations. Dr. Walter A. Jayne, of Denver, read an interesting paper entitled the clinical examination of blood. This is a subject which should engage the attention of physicians in an increased degree.

This volume of transactions takes rank among the leading state society publications.

---

A Manual of Diseases of the Ear, for the Use of Students and Practitioners of Medicine. By Albert H. Buck, M. D., Clinical Professor of the Diseases of the Ear, College of Physicians and Surgeons, Columbia College, New York; Consulting Aural Surgeon, New York Eye and Ear Infirmary and the Presbyterian Hospital. Second revised edition. Illustrated. Small 8vo, pp. x.—458. Price, cloth, $2.50. New York: William Wood & Co. 1895.

The first edition of this manual was published in 1889 and reviewed by the Journal of that year (Vol. XXVIII., 1888-9, p. 772). The book received general professional favor and a second edition is now presented, in which a new chapter has been introduced, entitled Analysis of Symptoms, some additional text has been added to the chapter on Chronic Purulent Inflammation of the Middle Ear, and the section devoted to a description of operations upon the mastoid process has been entirely rewritten and very much amplified. In its present form the student has a trustworthy guide to the diagnosis and treatment of diseases of the ear, written by an experienced and conservative teacher and practitioner.

The previous review gives an ample description of this work and we need not repeat it here. But we desire to say, as was said then, that it "deserves unqualified praise." A. A. H.

---

Notes on the Newer Remedies, their Therapeutic Applications and Modes of Administration. By David Cerna, M. D., Ph. D., Demonstrator of Physiology and Lecturer on the History of Medicine in the Medical Department of the University of Texas; formerly Assistant in Physiology, Demonstrator of, and Lecturer on, Experimental Therapeutics in the University of Pennsylvania, etc., etc. Small 8vo, pp. 253. Second edition, enlarged and revised. Price, $1.25. Philadelphia: W. B. Saunders, 925 Walnut street. 1895.

The first edition of this book was noticed in the Journal for November, 1893. It now appears thoroughly revised, with many of its articles rewritten, thus bringing it forward to the end of 1894. The object of the book is to keep brief records of the applications of the newer remedies, especially those whose usefulness have stood the test of clinical investigation. New features of the present edition are paragraphs on physiological action and toxicology, as well as those on incompatibility and contra-indications. An index of diseases has been added in the interest of the general practitioner of medicine. It is an excellent little book of reference.

---

Catalogue of Stoddart Bros., 84 Seneca street, Buffalo, N. Y., importers, manufacturers, wholesale and retail dealers in surgical instruments, trusses, braces, supporters, elastic stockings, batteries, crutches, operating tables, surgical chairs, orthopedic appliances, pharmaceutical preparations, strictly pure drugs and fine chemicals. Buffalo: Baker, Jones & Co. 1895.

The enterprising house of Stoddart Brothers is well known to every physician in Buffalo and the surrounding country. In addition to their large and extensive drug establishment, where everything wanted by either the physician or druggist can be obtained at wholesale or retail, they have the most complete surgical instrument department, for the manufacture, repair or sale of every sort and kind of instrument or surgical appliance, between New York and Chicago. This catalogue, which is well illustrated, is convenient for reference, and bespeaks a thrift and enterprise which might be expected by everyone who enjoys the personal acquaintance of the courteous proprietors. Those who do not happen to know them should make haste to visit their establishment, where they are sure to find everything wanted either in the drug or instrument line.

THE YEAR-BOOK OF TREATMENT FOR 1895. A Comprehensive and Critical Review for Practitioners of Medicine and Surgery. In one 12mo volume of 501 pages. Cloth, $1.50. Philadelphia: Lea Brothers & Co. 1895.

This book makes its eleventh annual appearance with reasonable promptitude. It is convenient to have the novelties of the year set in accessible form for reference and so arranged alphabetically that each subject sought may readily be found. This book conforms to its usual fashion in style and size and will be welcomed by all those who possess the previous volumes, as well as by a large number of recently made doctors who are anxious to keep pace with whatever is new and valuable in the practice of medicine. Whenever a searcher desires more complete information on any subject, he will find an excellent selected list of new books added at the end of the volume, that will enable him to obtain the information sought. The book possesses the merit of cheapness and usefulness combined.

---

BREAD FROM STONES. A New and Rational System of Land Fertilization and Physical Regeneration. Translated from the German. Duodecimo, pp. 135. Price, 25 cents. Philadelphia, Pa.: A. J. Tafel, 1011 Arch street. 1894.

This is a neatly printed little book of 135 pages. It is claimed that if the theories propounded by the author of the volume before us were put in practice in this country, it would free the farmer from a heavy yearly expense for fertilizer; at the same time gradually bring back his exhausted fields to their original state and produce food on which health might be maintained.

J. A. M.

---

### BOOKS RECEIVED.

Medical Gynecology. A Treatise on the Diseases of Women from the Standpoint of the Physician. By Alexander J. C. Skene, M. D., Professor of Gynecology in the Long Island College Hospital, Brooklyn, N. Y.; formerly Professor of Gynecology in the New York Post-Graduate Medical School; Gynecologist to the Long Island College Hospital, etc., etc. Octavo, pp. vi.—529. With illustrations. New York: D. Appleton & Co. 1895.

Report of the Commissioner of Education for the Year 1891–2. In two volumes. Washington: Government Printing Office. 1894.

Annual Report of the State Board of Charities for the Year 1894. Transmitted to the Legislature March 25, 1895. Albany: James B. Lyon, State Printer. 1895.

The Treatment of Wounds, Ulcers and Abscesses. By W. Watson Cheyne, M. B., F. R. S., F. R. C. S., Professor of Surgery in King's College, London. In one 12mo volume of 207 pages. Cloth, $1.25. Philadelphia: Lea Brothers & Co. 1895.

Twentieth Annual Report of the Secretary of the State Board of Health of the State of Michigan for the fiscal year ending June 30, 1892. Lansing: Robert Smith & Co., State Printers and Binders. 1894.

International Clinics. A Quarterly of Clinical Lectures on Medicine, Neurology, Surgery, Genito-urinary Surgery, Gynecology, Pharyngology, Rhinology, Ophthalmology, Laryngology, Obstetrics, Otology and Dermatology. By professors and lecturers in the leading medical colleges of the United States, Great Britain and Canada. Edited by Judson Daland, M. D. (Univ. of Penna.), Philadelphia, Instructor in Clinical Medicine and Lecturer on Physical Diagnosis in the University of Pennsylvania; Assistant Physician to the University Hospital; Physician to the Philadelphia Hospital and Fellow College of Physicians, Philadelphia. J. Mitchell Bruce, M. D., F. R. C. P., London, England, Physician to, and Lecturer on, Therapeutics at the Charing Cross Hospital. David W. Finlay, M. D., F. R. C. P., Aberdeen, Scotland, Professor of Practice of Medicine in the University of Aberdeen; Physician to, and Lecturer on, Clinical Medicine in the Aberdeen Royal Infirmary; Consulting Physician to the Royal Hospital for Diseases of the Chest, London. Volume I. Fifth Series. 1895. Royal 8vo, pp. xii.—354. Philadelphia: J. B. Lippincott Co. 1895.

Manual of General Medicinal Technology, Including Prescription Writing. By Edward Curtis, A. M., M. D., Emeritus Professor of Materia Medica and Therapeutics, College of Physicians and Surgeons, Medical Department of Columbia College, in the City of New York. Third edition, conforming to the U. S. Pharmacopeia of 1890. Pocket size (Wood's Pocket Manual Series), 245 pages. Price, $1.00.

Transactions of the Sixteenth Annual Meeting of the American Laryngological Association, held in the City of Washington, D. C., May 30, 31 and June 1, 1894. New York: D. Appleton & Co. 1895.

Eighth Annual Report of the Managers of the St. Lawrence State Hospital for the Year 1894. Albany: James B. Lyon, State Printer. 1895.

Ninth Annual Report of the State Board of Health and Vital Statistics of the Commonwealth of Pennsylvania. Clarence M. Busch, State Printer of Pennsylvania. 1894.

Cod Liver Oil and Chemistry. By Peckel Möller, Ph.D., London; Peter Möller, 43 Snow Hill, E. C. New York: W. H. Schieffelin & Co. 1895.

A Book of Detachable Diet Lists and a Sick-room Dietary. Compiled by Jerome B. Thomas, A. B., M. D. Price, $1.50. Published by W. B. Saunders, 925 Walnut street, Philadelphia. 1895.

Transactions of the Antiseptic Club. Reported by Albert Abrams, a Member of the San Francisco Medical Profession. Illustrated. Price, $1.75. New York: E. B. Treat, 5 Cooper Union. San Francisco: Johnson & Emigh. 1895.

Twentieth Century Practice. An International Encyclopedia of Modern Medical Science. By leading authorities of Europe and America. Edited by Thomas L. Stedman, M. D., New York City. In twenty volumes. Volume II. Nutritive Disorders. New York: William Wood & Co. 1895.

Index of Medicine. By Seymour Taylor, M. D., Member Royal College of Physicians; Senior Assistant Physician to the West London Hospital. In one large 12mo volume of 801 pages, with 35 engravings. Cloth, $3.75. Philadelphia: Lea Brothers & Co.

A Manual of the Modern Theory and Technique of Surgical Asepsis. By Carl Beck, M. D., Visiting Surgeon to St. Mark's Hospital and to the German Poliklinik of New York City, etc. Small 8vo, pp. 300. With sixty-five illustrations in the text and twelve full-page plates. Price, $1.25 net. Philadelphia: W. B. Saunders, 925 Walnut street. 1895.

La Pratique des Maladies du Cœur et de L'Appareil Circulatoire dans les Hopitaux de Paris. Aide-Mémoire et Formulaire de Thérapeutique Appliquée par Le Professeur Paul Lefert. Paris: Librairie J. B. Bailliére et Fil, Rue Hautefeuille, 19 près le Boulevard Saint-Germain. 1895.

Urinalysis, Including Blanks for Recording the Analysis and Microscopic Examination of the Urine. For Medical Practitioners, Life Insurance Examiners and Specialists. Arranged by Joseph C. Guernsey, A. M., M. D. Philadelphia: J. B. Lippincott Co. 1895.

